# Performance of large language artificial intelligence models on solving restorative dentistry and endodontics student assessments

**DOI:** 10.1007/s00784-024-05968-w

**Published:** 2024-10-07

**Authors:** Paul Künzle, Sebastian Paris

**Affiliations:** Department of Operative, Preventive and Pediatric Dentistry, Charité – Universitätsmedizin Berlin, Aßmannshauser Str. 4-6, Berlin, 14197 Germany

**Keywords:** ChatGPT, Gemini, Natural language processing, Artificial intelligence, GenAI

## Abstract

**Objectives:**

The advent of artificial intelligence (AI) and large language model (LLM)-based AI applications (LLMAs) has tremendous implications for our society. This study analyzed the performance of LLMAs on solving restorative dentistry and endodontics (RDE) student assessment questions.

**Materials and methods:**

151 questions from a RDE question pool were prepared for prompting using LLMAs from OpenAI (ChatGPT-3.5,-4.0 and -4.0o) and Google (Gemini 1.0). Multiple-choice questions were sorted into four question subcategories, entered into LLMAs and answers recorded for analysis. P-value and chi-square statistical analyses were performed using Python 3.9.16.

**Results:**

The total answer accuracy of ChatGPT-4.0o was the highest, followed by ChatGPT-4.0, Gemini 1.0 and ChatGPT-3.5 (72%, 62%, 44% and 25%, respectively) with significant differences between all LLMAs except GPT-4.0 models. The performance on subcategories direct restorations and caries was the highest, followed by indirect restorations and endodontics.

**Conclusions:**

Overall, there are large performance differences among LLMAs. Only the ChatGPT-4 models achieved a success ratio that could be used with caution to support the dental academic curriculum.

**Clinical relevance:**

While LLMAs could support clinicians to answer dental field-related questions, this capacity depends strongly on the employed model. The most performant model ChatGPT-4.0o achieved acceptable accuracy rates in some subject sub-categories analyzed.

## Introduction

Artificial intelligence is the modelling of human intelligence by automated machines and holds exceptional potential in healthcare and its industry to advance patient care. Natural language processing (NLP), the ability of computers to understand and process human language, could assist making human-computer interaction more efficient and convenient. Large language model (LLM)-based artificial intelligence (AI) applications (LLMAs), which use NLP techniques to generate answers to text-based input, have gained particular attention since the release of OpenAI’s ChatGPT in November 2022 [1]. Subsequently, in February 2023, the chat-based LLMA Google Gemini, then called Bard, was released for the public [2]. The term generative artificial intelligence (GenAI) is used to describe their ability to generate texts, while other GenAI applications can also generate videos and images. There has been a rising number of patients and professionals who turn to these publicly available LLMAs to find answers for diverse questions, including those on healthcare.

In the history of medicine and dentistry, the ability to perform certain practical techniques and, above all, the medical knowledge of practicing doctors have determined their ability to diagnose diseases and to heal patients, i.e. to be a “good” physician or dentist. Consequently, during the studies of medicine and dentistry, tremendous amounts of factual knowledge are learned by students, which is an essential part of medical examinations. The exclusivity of knowledge to medically educated persons has always limited the access to good medical treatment for many patients. With LLMAs, artificial intelligence becomes available to dentists and patients alike and covers the areas of spoken and written language. The tremendous popularity of these applications across disciplines has seen massive leverage potential and use across various medical specialties [3–14].

In dentistry, the potential uses of LLMAs were evaluated and include decision support for clinical situations, patient and dental education and support with scientific writing and scientific education [15]. To support decisions and to evaluate their capacity to serve as a prospective adjuvant tool in clinical decision making, ChatGPT-4.0 and ChatGPT-3.5 were prompted to assist in the management of odontogenic sinusitis cases. While the LLMAs tested provided some assistance, they are not ideal to aid clinical management support [16]. To examine its use in patient education, ChatGPT was used to provide answers for patients that expect a third molar extraction. While answers were mostly correct, the readability was poor and difficult to understand for the average patient [17]. For the evaluation of its use in dental education, it was shown that ChatGPT already lends itself for students to create a “personalised learning experience” [18], to help with writing dental essays and for educators to create test questions [19]. In dental scientific writing, ChatGPT has already seen a massive increase in usage [20] and could further increase research productivity [21]. For example, a LLMA was already used successfully for the draft of readable cover letters [22, 23].

At present, a shortage of studies that thoroughly examine the performance of LLMAs on dental questions, and restorative dentistry and endodontics (RDE) test questions in particular, stand in the way of any understanding of the practical use and potential risks for exam preparation and knowledge mining in dentistry. For example, it remains unknown how the use of artificial intelligence will impact academic integrity and university education. LLMAs can be used for knowledge mining by e.g. students, however, the LLMAs are prone to hallucination. This term describes their habit to convincingly state false information, which hence requires specific caution during the use of such LLMAs [24]. In science, the use of artificial intelligence is condemned and ChatGPT must not be listed as a scientific author due to its inability to account for and approve manuscript submissions [25–28]. Moreover, the performance of different LLMAs is unknown, and furthermore, whether some applications lend themselves more to professional use than others. It remains to be discovered whether LLMAs can convincingly answer questions from a RDE question pool. Moreover, it is of particular interest whether these applications could be used by students to find answers during their studies or clinical practice after graduation.

This study focused on the ability of LLMAs to support RDE clinical decision-making and knowledge mining by analyzing their capacity to answer test questions from a RDE question pool. The null hypothesis analyzed was that all LLMAs assessed do not show statistically different percentages of correct answers to the question bank of RDE test questions. We examined and compared the performance of four different LLMAs ChatGPT-3.5, -4.0, -4.0o and Google Gemini 1.0 and investigated their relative ability to answer questions across different subspecialties of RDE. Lastly, it was studied whether some LLMAs may express subject-specific advantages and are therefore more applicable for usage than other LLMAs in some subspecialties of dentistry.

## Methods

### Database for clinical questions

**Fig. 1:**
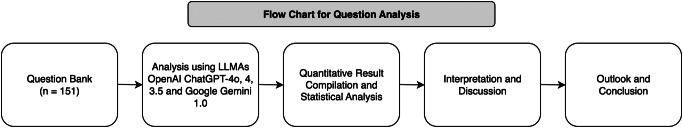
Flowchart for question analysis using four different LLMAs. Questions were selected from a RDE test question pool and subsequently subject to analysis. Questions were entered into the text field of the respective LLMA, and answers systematically collected. Afterwards, the results were compiled and statistically interpreted

Questions were retrieved from a question pool of RDE test questions from the Department of Operative, Preventive and Pediatric Dentistry at Charité – Universitätsmedizin Berlin (for a flowchart, see Fig. 1). The question database is continuously updated with new questions and organized into four different question pools: direct restorations, endodontics, indirect restorations, and caries.

For validity purposes, image-based questions and prose questions were excluded from the analysis. Brackets with points to be allocated in exams were removed prior to the questions being included in the question database. Only multiple-choice questions with one single correct answer were used for this study. A total of 159 questions were available, of which 8 questions were excluded due to missing or wrong answer options. 151 questions were used for further analyses. Two types of questions were used. To answer Type-A-questions (*n* = 145), the right answer option that includes the correct combination of statements referring to the question had to be selected. The question type was used in all subcategories. For Type-B-questions, a single correct answer statement had to be chosen. Type-B-questions (*n* = 6) were present only in the subcategory direct restorations. Due to the different complexity of question types and a potential impact on LLMA performance, an additional analysis was conducted that differentiates between question types.

### Data management

The prompting for the LLMAs Gemini 1.0, ChatGPT-3.5 and 4.0 was performed between January 20th and February 8th, 2024. ChatGPT-4.0o prompting was performed between May 15th and 24th, 2024. The prompting period was kept to a minimum. All questions were individually entered by one investigator into ChatGPT-3.5, -4.0, -4.0o and Google Gemini 1.0 using their websites (https://chat.openai.com/?model=text-davinci-002-render-sha, https://chat.openai.com/?model=gpt-4, https://chatgpt.com/?model=.

gpt-4o and https://gemini.google.com/app) in German language with an English prompt. Before entering a new question, a fresh session was started by deleting prior chats and thereby decreasing the chance of information overflow and memory retention bias. Each question was entered once only during the analysis. To ensure result quality and generalizability of results, one prompt for all LLMAs and questions was used to let the LLMA choose the correct answer of different options. There were two different question types used, question type A for most questions and question type B in some instances of questions on direct restorations: The questions were designed for clinical stage dental students learning RDE with a passing threshold of 60%. The answer of the LLMA was chosen which it deemed correct or most correct. If the LLMA did not give a distinct answer, the answer it considered most correct was selected for analysis. All answers from all LLMAs were subsequently put into a Microsoft Excel (version 16.88) datasheet for comparison to correct answers. Total, subject sub-category and question-type based result performance were calculated.

### Statistical analysis

The LLMAs were tested using chi square and p value analyses using the Bonferroni correction in Python (version 3.9.16.).

## Results

**Fig. 2:**
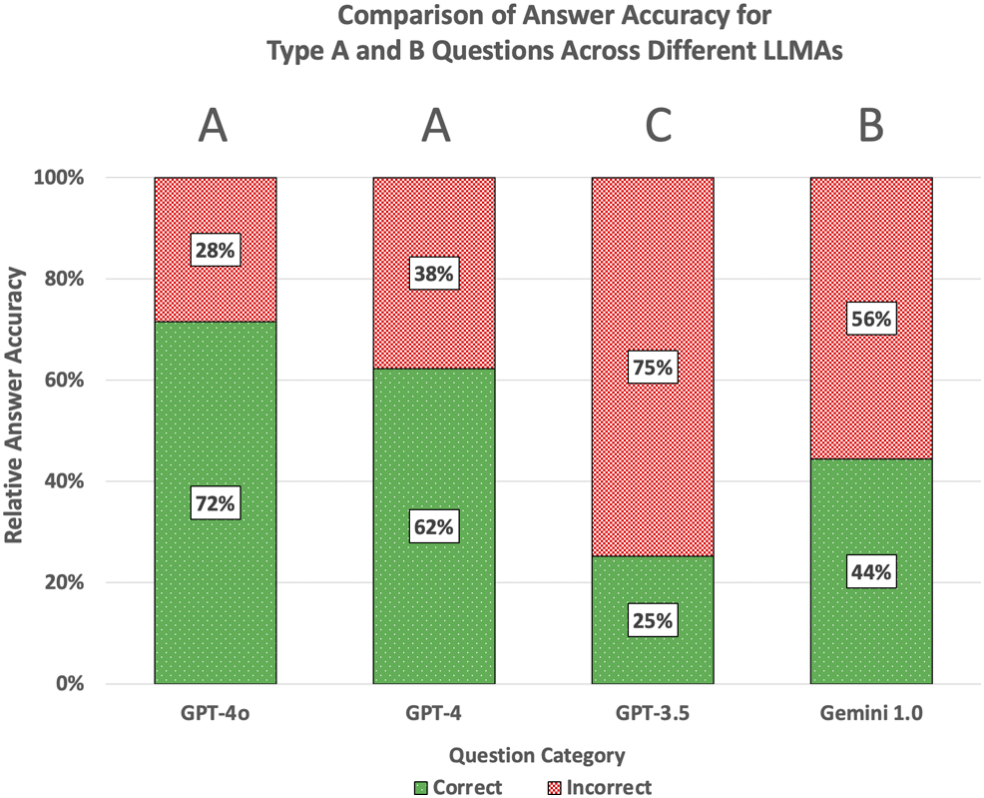
Relative answer accuracy of different LLMAs. For each LLMA, the percentage of correct answers is shown by a green bar, while incorrect answers are displayed as red bars. Significant differences between the LLMAs are indicated with different letters (Chi^2^; *p* < 0.001 for all significant differences between LLMAs except GPT-3.5 and Gemini 1.0 (*p* < 0.01)). LLMAs are labelled using short forms of their respective names (GPT-4.0o: OpenAI ChatGPT-4.0o, GPT-4: OpenAI ChatGPT-4.0, GPT-3.5: OpenAI ChatGPT-3.5, Gemini 1.0: Google Gemini 1.0) and sorted by their developer and release date (OpenAI GPT-4.0o: May 13, 2024; GPT-4.0: March 14, 2023; GPT-3.5: November 30, 2022; Google Gemini 1.0: December 15, 2023)

Overall, 151 questions were used for analysis, of which correctly answered were 108 questions by ChatGPT-4.0o (72%), 94 questions by ChatGPT-4.0 (62%), 67 questions by Google Gemini 1.0 (44%) and 38 questions by ChatGPT-3.5 (44%). Relative answer accuracy is depicted in Fig. 2. ChatGPT-4.0o and ChatGPT-4.0 performed statistically better (*p* < 0.001) compared to all other LLMAs but the difference between both did not reach significance level (*p* = 0.0844).

**Fig. 3:**
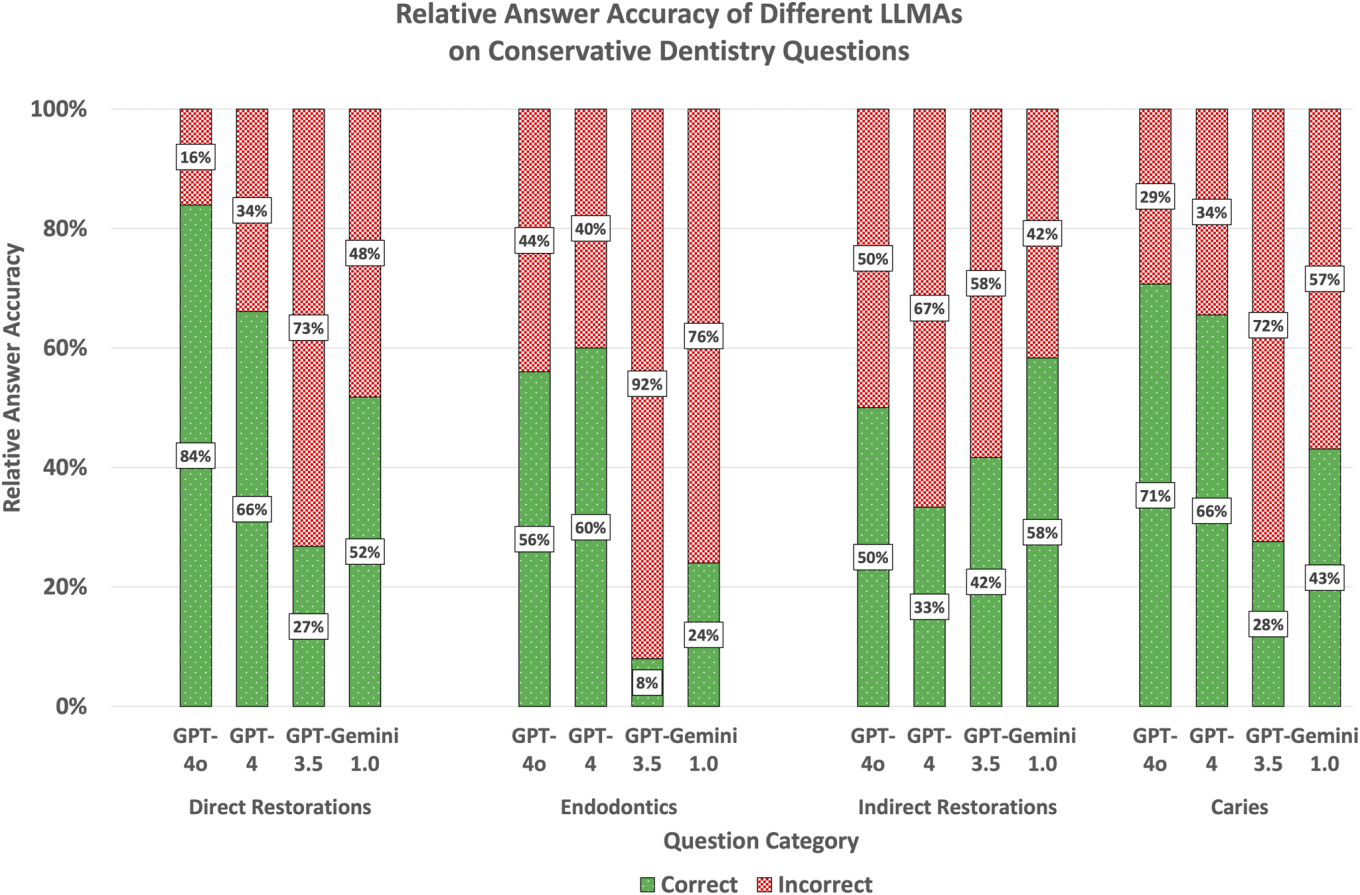
Relative answer accuracy of different LLMAs on RDE question categories. For each individual subject direct restorations (*n* = 56), endodontics (*n* = 25), indirect restorations (*n* = 12) and caries (*n* = 58), individual stacked bar charts indicate the relative share of correctly (green) and incorrectly (red) answered test questions. The average performance rate across all LLMAs assessed was best for the subcategory direct restorations (48%), followed by caries (45%), indirect restorations (44%) and endodontics (31%)

**Fig. 4:**
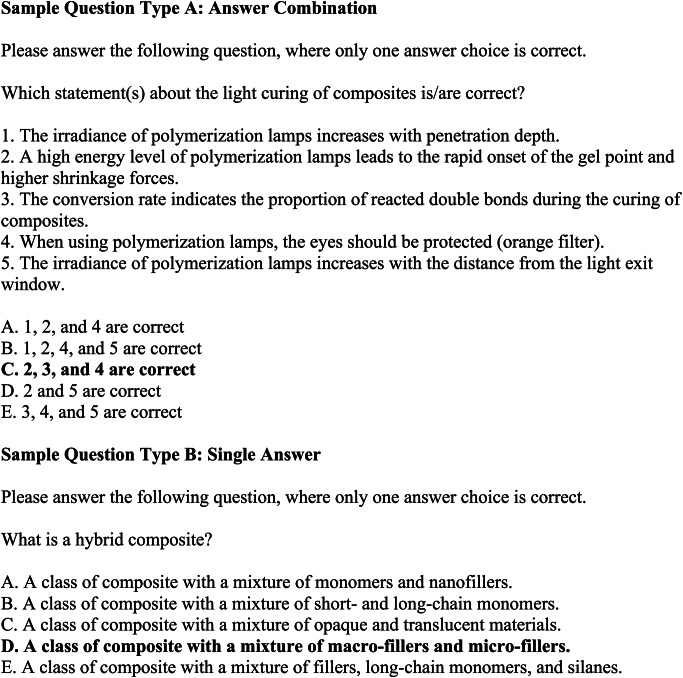
Sample question prompt types used for the assessment. Quantitative questions were entered into the LLMA using the same prompt, but different answer choices. In style A, the LLMA had to select an answer choice that refers to statements made for the question asked. Style B directly asked for the correct answer choice for the question posed. Correct answers are marked in bold font

**Fig. 5:**
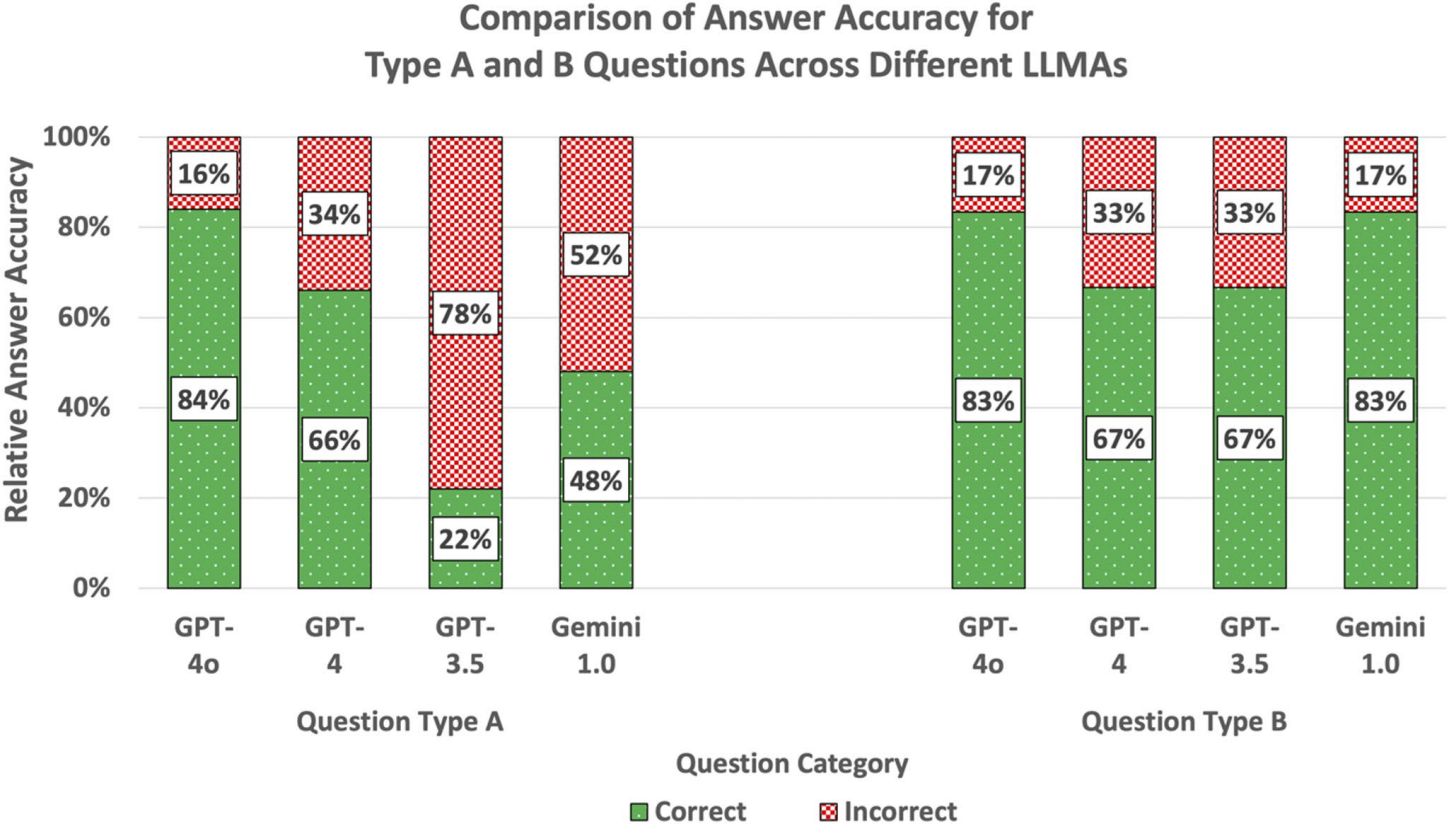
Answer accuracy sorted by question type in subcategory direct restorations. In the subcategory direct restorations, *n* = 50 questions of type A and *n* = 6 questions of type B were compared across different LLMAs

**Fig. 6:**
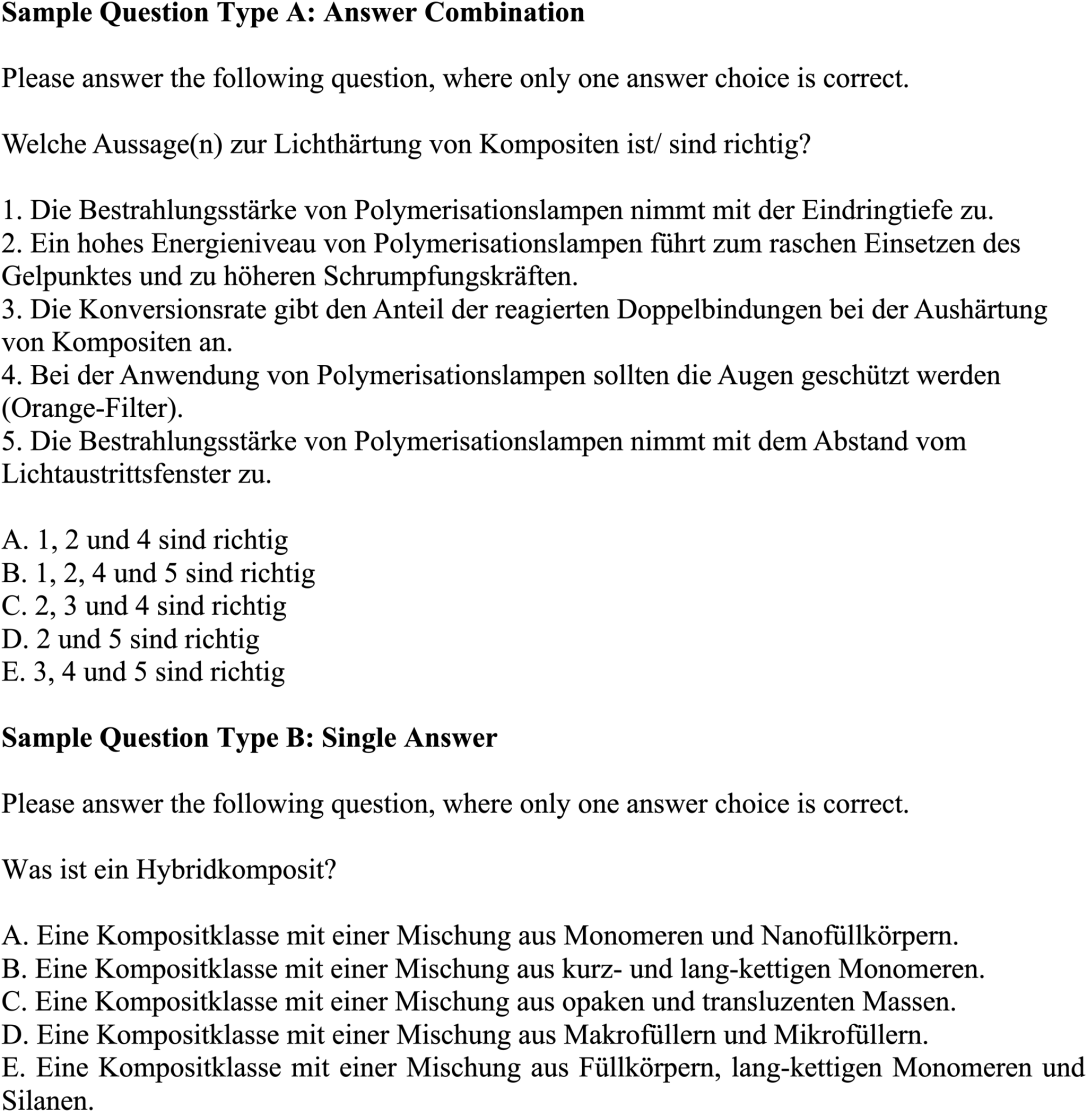
Examples of verbatim question prompts used in LLMAs. The questions used in prompts were kept in German as the original language for the assessment. The prompt for the application was phrased in English.

Sorted by the different thematic categories and in descending order, the answer accuracy of ChatGPT-4.0o was the highest of all LLMAs in subcategories direct restorations (84%) and caries (71%) and was marginally trumped by GPT-4.0 in endodontics (60%) and Gemini 1.0 for indirect restorations (58%). ChatGPT-4.0 was good at answering questions from categories caries (66%) and direct restorations (66%), slightly less at endodontic questions (60%, but still the most performant LLMA in this category) and performed poor on questions on indirect restorations (33%). ChatGPT-3.5 was the worst performing LLMA for all subcategories (direct restorations: 27%, endodontics: 8%, caries: 28%) except for indirect restorations (42%), where ChatGPT-4.0 performed the poorest (33%). In endodontics, its performance was worse than the average statistical performance rate if answer choices were guessed (20 or 25%, depending on the number of answer choices). Google Gemini 1.0 showed its highest answer accuracy for indirect restorations (58%), where it was the most performant LLMA. In all other categories, the LLMA performed worse than ChatGPT-4.0o and -4.0 (direct restorations: 52%, endodontics: 24%, caries: 43%). The answer accuracy for individual questions across different subjects and LLMAs is shown in Fig. 3. Sample questions of Type A and B are depicted in Fig. 4. The answer accuracy was on average higher for questions phrased in style B compared to style A questions. A comparison of answer accuracy of different test question styles A and B is provided in Fig. 5. Verbatim question prompts used in LLMAs are shown in Fig. 6.

## Discussion

So far, there has been only limited data on the availability of GenAI-derived dental information. This study systematically evaluated the performance of chat-based LLMAs on RDE questions. While these LLMAs could prove to be a useful assistant in dental education, AI tools cannot replace conventional modes of teaching at present. What should be regarded with caution is that ChatGPT can answer any question with bold conviction, even when it is wrong [24]. Moreover, the application was shown to provide fake references in the past [29, 30].

Early in the process, medical tests such as the USMLE were used as a test model and ChatGPT was found to be passing or almost passing all three stages of the exam [31]. A closer examination of the performance of ChatGPT-4.0 and ChatGPT-3.5 on the exam’s part 3 showed that the more advanced version ChatGPT-4.0 (84.7%) was performing significantly better than its predecessor ChatGPT-3.5 (56.9%), which was also found in our findings [7]. A study using dermatology specialty certificate examinations from Poland found accuracy rates between 60.5% and 68.9% for ChatGPT-3.5 and 80.7% and 84.0% for ChatGPT-4.0 [13]. In an evaluation of the Taiwanese Family Medicine Board Examination, ChatGPT-3.5 noticeably failed the exam with a correctness rate of only 41.6%, which is in line with our finding that the free version of ChatGPT delivers only mediocre results on dental questions [32]. In an examination of otolaryngology questions, the combined accuracy across all question types and subcategories assessed using ChatGPT-3.5 was 63%, 19% higher than the LLMA’s performance on the questions used in our study, which is likely related to the question phrasing used [3]. Where the question style involved multiple correct answers, the accuracy dropped to 34%. This result and question style is corresponding more closely with results for question type A used in this study, involving multiple correct answer statements [3]. In an assessment of sleep medicine questions, similar accuracy rates for ChatGPT-3.5 (46.8%), ChatGPT-4.0 (68.1%) and Google Bard (now Gemini, 45.5%) were found as in our study. The assessment of Taiwan plastic surgery board questions showed accuracy rates of 59% for ChatGPT-4.0 compared to 41% for ChatGPT-3.5, also finding similar performance rates as our study [33]. For ChatGPT-4.0o, an analysis using pediatric nephrology questions found a total performance rate of 75.2%, corresponding well to the LLMA’s performance found in this study (72%) [34].

Moreover, the LLMAs were assessed in different dental question settings, i.e. the Japanese National Dentist Examination [35], prosthodontic questions [36], the identification of predatory dental journals [37] or dental amalgam questions [38]. A Japanese study evaluated the performance of GPT-3.5, -4.0 and Gemini 1.0 on questions of the Japanese National Dental Examination and found a collective performance rate of 51.9% for GPT-3.5, 66.5% for Google Bard (today: Gemini) and 73.5% for GPT-4.0 [35]. These performance rates are considerably higher than the success rates found in this study, which discovered 26.9% (GPT-3.5), 22.5% (Gemini 1.0) and 11.5% (GPT-4.0) less performance for the respective LLMAs. The most recent LLMA ChatGPT-4.0o was not assessed but comes close to the reported results with an accuracy rate of 72%. In an assessment with dental head and neck anatomy questions, ChatGPT-3.5 was able to answer 73.33% of the questions correctly [39], a notably higher performance rate than our study. Endodontology questions were examined in a dichotomous question format with a total accuracy rate of 57.33% of ChatGPT-3.5, much higher than the findings of our study [40]. There, the question format, asking for a simple “yes” or “no”, has likely assisted the LLMA to answer correctly.

In the present study, the most advanced and latest GPT version was also the most performant model. Since LLMAs improve over time, which was exemplified by the overall performance increase from the earlier model ChatGPT-3.5 over ChatGPT-4.0 and the latest model ChatGPT-4.0o in the literature available and in the present study, future models may become even more applicable in dental education and a more reliable tool for knowledge mining. An impressive example of the rapid evolvement and performance increase over time of ChatGPT is shown by the British Overseas Registration Examination (ORE), which is divided in Part 1 and 2 for written and clinical components, respectively. An early study of the performance of ChatGPT of its performance showed that it could only pass the first part of the written examination (Paper A) but failed the second written exam (Paper B) [41]. ChatGPT-4o, the latest publicly available model of OpenAI, also passes Paper B [42]. While this increase in performance was promising and underscores the future potential of LLMAs, many barriers still need to be overcome before a regular application in clinical practice or education become feasible. A full integration into academic teaching, research and clinical practice, while conceivable, will require considerable increases in performance and reliability. Before LLMAs become integrated into regular academic practice, however, risks of automation bias and overreliance on LLMAs need to be thoroughly considered.

It remains to be discussed why some LLMAs perform better than others in certain medical subspecialties or dental subcategories as shown in the present study. For one, the dental training data used may not be as sophisticated and are not publicly available. While it can only be suspected that the data used to train the LLMAs did not contain dental questions to a similar extent as medical questions, this may be due to an availability bias of publicly available dental questions, which is the result of a smaller student body and narrower institutionalization of the profession compared to medicine. By reducing the siloing of dental data, available information could be freed for use in dental training datasets and enhance currently undertrained LLMAs. The poor performance specifically of ChatGPT-3.5 on endodontic questions could be explained by the complexity of the variables that are considered before a root canal treatment, which may have been part of the vast training data sets used, but could have contained contradictory information given the vast developmental changes in the field over the past decades [43].

Moreover, the LLMAs may need further cycles of reinforcement learning. It becomes evident that the more advanced LLMAs may have undergone more sophisticated reinforcement learning cycles that made the LLMAs more knowledgeable and increased its potency to answer dental test questions. In future applications, however, ChatGPT may become a clinical healthcare assistant which advises dental treatment decisions of both patients and dental professionals. This could be particularly relevant for decisions that require to draw correct inferences from multiple patient findings, as in endodontic diagnostics. In further analyses, research on prompt engineering could identify optimization potential for the way RDE questions are entered into the application. The analysis of individual question responses by ChatGPT-4.0o revealed that the LLMA does show weaknesses in specific subcategories, which are predominantly due to incorrect or contradicting information of the LLMA. In some instances, the LLMA correctly answered questions that were imprecisely formulated and revealed potential for clarification. In the academic field, this demeanor could therefore make LLMAs applicable towards the identification of vaguely phrased questions in academic exams and improve question quality.

Future analyses should let LLMAs answer prose-based questions, which were excluded in this analysis. This would require more sophisticated and individualized prompts which were not part of this study. Specifically, these prompts would require LLMAs to e.g. fill in one line only, decipher acronyms, or fill in blank spaces. Essay-type dental questions were successfully entered into a version of ChatGPT previously, yet it required an adapted methodology. There, the LLMA performed well, yet less positively compared to multiple choice questions [44]. Moreover, for a precise evaluation of LLMAs and for a true comparison to student performance, image-based questions would need to be answered by these applications as well. After modification of image-based questions to fit the entry requirements of LLMAs, the performance of LLMAs should be evaluated as well and a full comparison between human (i.e. student) and AI performance on dental tests can be conducted.

The questions used for the assessment of LLMAs in this study were sourced from one of the largest teaching hospitals in Europe, Charité – Universitätsmedizin Berlin and were not artificially generated. The performance results for RDE test questions therefore have extended applicability but should be regarded critically since they were retrieved from one teaching location and country only, which has country- and university-specific teaching requirements. To extend generalizability, further studies should include more diverse question sets from different clinics and countries. Moreover, if the LLMA knew the location where the question was asked, it could have included that information and/or country-specific expert guidelines, which should be analyzed in future research.

Questions were asked in German with an English prompt to reduce translation biases. Although LLMAs are well trained for translations between different languages and an examination using an original (Polish) and English language found no significant differences in performance between question sets phrased in different languages, this may have affected the result outcome [13].

Overall, the sample size of 151 questions is not as large as sophisticated question banks that were assessed in other analyses previously. However, these question banks often cover various topics and have similar question bank sizes if only one topic/specialty is regarded. Among subject subcategories, the subcategories endodontics and indirect restorations were underrepresented. Specifically, indirect restorations made up only 12 of 151 questions in total, which may limit their result validity. Moreover, the question type B contained only *n* = 6 questions and was only represented in the subcategory direct restorations, which marks an availability bias. This can be explained by a faculty preference for using questions that test for a deeper understanding of subject matter of students using answer combinations in question type A. Moreover, the question scope of RDE only allows constrained statements on the general performance of LLMAs on dental questions.

Before entering each question, a new session was started to reduce information overflow from previous questions. However, due to the continuous learning of the LLMA, it is not known whether the entry of questions can impact the performance on subsequently entered questions even using a fresh session. In addition, LLMAs can adapt with every prompt entered and learn longitudinally over time. While we reduced the timeframes for prompting to a minimum, this learning behavior could have had an influence on our results, although the difference in outputs was found to be limited in a study involving a one-week cadence between prompts [38]. Further investigations could assess the development of the performance of LLMAs and their longitudinal performance development on RDE test questions.

## Conclusions

The analyzed LLMAs from OpenAI (ChatGPT-3.5,-4.0 and -4.0o) and Google (Gemini 1.0) showed very different performance rates. Over the entire range of RDE student assessments, ChatGPT-4.0o was the most performant, followed by ChatGPT-4.0, Gemini 1.0 and ChatGPT-3.5. In the specific RDE subcategories, ChatGPT-4.0o was the most performant in subcategories direct and indirect restorations as well as caries, while ChatGPT-4.0 was the most performant in endodontics. Overall, the ChatGPT-4 models were the most performant and lend themselves the most towards cautious use in RDE knowledge mining and dental education.

## Data Availability

Due to legal data protection reasons, the data used in this analysis are not publicly available. Data used for this study are available from the corresponding author on reasonable request.
